# Human Skin Hypoxia Modulates Cerebrovascular and Autonomic Functions

**DOI:** 10.1371/journal.pone.0047116

**Published:** 2012-10-08

**Authors:** Olivia Pucci, Clifford Qualls, Anne Battisti-Charbonney, Dahlia Y. Balaban, Joe A. Fisher, Jim Duffin, Otto Appenzeller

**Affiliations:** 1 Department of Anesthesiology, University of Toronto, and University Health Network, Toronto, Ontario, Canada; 2 Institute of Medical Science, University of Toronto, Toronto, Ontario, Canada; 3 Department of Mathematics and Statistics, University of New Mexico, Albuquerque, New Mexico, United States of America; 4 Department of Physiology, University of Toronto, Toronto, Ontario, Canada; 5 Department of Neurology, New Mexico Health Enhancement and Marathon Clinics Research Foundation, Albuquerque, New Mexico, United States of America; Université de Montréal, Canada

## Abstract

Because the skin is an oxygen sensor in amphibians and mice, we thought to confirm this function also in humans. The human upright posture, however, introduces additional functional demands for the maintenance of oxygen homeostasis in which cerebral blood flow and autonomic nervous system (ANS) function may also be involved. We examined nine males and three females. While subjects were breathing ambient air, at sea level, we changed gases in a plastic body-bag during two conditions of the experiment such as to induce skin hypoxia (with pure nitrogen) or skin normoxia (with air). The subjects performed a test of hypoxic ventilatory drive during each condition of the experiment. We found no differences in the hypoxic ventilatory drive tests. However, ANS function and cerebral blood flow velocities were modulated by skin hypoxia and the effect was significantly greater on the left than right middle cerebral arteries. We conclude that skin hypoxia modulates ANS function and cerebral blood flow velocities and this might impact life styles and tolerance to ambient hypoxia at altitude. Thus the skin in normal humans, in addition to its numerous other functions, is also an oxygen sensor.

## Introduction

The skin is the first tissue to encounter changes in the environment. Not surprisingly, it reacts with speed to alterations, such as hypoxia, that might threaten the survival of the organism, and the brain is especially sensitive to hypoxia. But reactivity to immediate environmental threats is too important to be entrusted to volitional control. Thus, the autonomic nervous system (ANS), a part of the nervous system that is largely independent of volition, might be brought into service to provide the necessary counter-measures to maintain homeostasis.

To test the hypothesis that the skin is an oxygen sensor in humans we examined the cerebral circulation and ANS function in humans exposed to regional skin hypoxia.

We examined nine males and three females. While subjects were free to breathe room air, at sea level, we changed gases in a plastic body-bag that, nevertheless, allowed unrestricted access through the mouth and nose to room air.

We changed conditions in the body bag during the experiments such as to induce regional skin hypoxia (with pure nitrogen) or leave the skin normoxic (with room air). The subjects performed a test of hypoxic ventilatory drive during each condition of the experiment.

**Figure 1:**
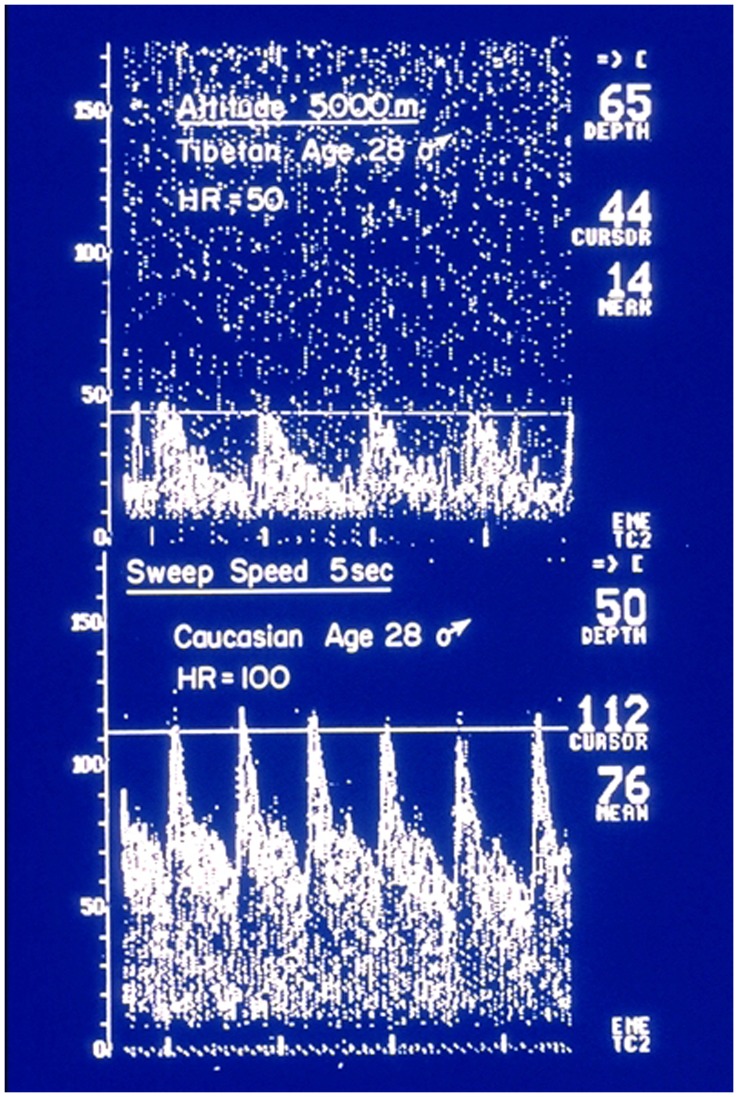
Middle cerebral artery flow velocities recorded in the field from the left artery in one Tibetan age 28 and one sea level Caucasian of the same age at 5000 m. in the Himalayas. Acute altitude exposure for the Caucasian one day after arrival at the Everest base camp on the Tibetan side of the mountain. Note the differences in heart rate and flow velocities in these healthy young subjects. (from reference #3, with permission).

The skin does not rely on the circulation alone for its oxygen supply. For example, when ambient air becomes hypoxic, as on altitude sojourn, skin constituent cells (keratinocytes) release nitric oxide which induces skin vasodilatation and thus improves the supply of oxygen to the skin by an increase in blood flow [1]. A similar response to ambient hypoxic exposure is found in the human brain [2]. This marked increase in cerebral blood flow is evident on acute exposure to altitude in sojourners shown in Doppler recordings (Fig. 1) but this reverses to normal after several days at altitude. A similar response to ambient hypoxic exposure is found in the human brain [2]. Our short lasting experiments are consistent with this. The exact mechanism of the initial vasodilatation remains controversial [3] but nitric oxide release from the cerebrovascular endothelium is a likely agent of change in this setting too.

Studies in mice have shown that the skin may act as a “coordinator” of the whole organism’s responses to hypoxia” by modulating blood flow through the skin [4]. Thus an important response to hypoxia may be controlled by fur-covered skin in mice. Also the hairless skin of amphibians is crucial for the systemic responses to hypoxia in these vertebrates [4].

In humans, the skin is usually covered by clothing which serves to coordinate responses to acute temperature variations using changes in clothing-type and materials; in this setting skin responses to hypoxia are influenced by its artificial coverings. Skin function in response to the environment is also affected by modern lifestyle which may impact human physiologic reactions to hypoxia. The role of the skin in human adaptation to hypoxia remains largely unexplored, but it may also play an important role in survival in ambient hypoxia in the mountainous regions of the world.

Here we show that the normal human skin responds to acute hypoxia by modulating the cerebral circulation and ANS function to maintain homeostasis.

## Results

### Chemoreflex Control of Breathing

The control of breathing by respiratory chemoreflexes is part of the reflex control mechanisms that maintain arterial partial pressures of carbon dioxide (Pco_2)_ and oxygen (Po_2_).

There were no statistically significant differences in the chemoreflex control of breathing in the two conditions of this experiment (skin normoxia or skin hypoxia). No significant differences between the two test conditions of the experiment, in skin temperature, heart rate and pulse oximeter records, obtained by surface sensors, were found.

**Figure 2:**
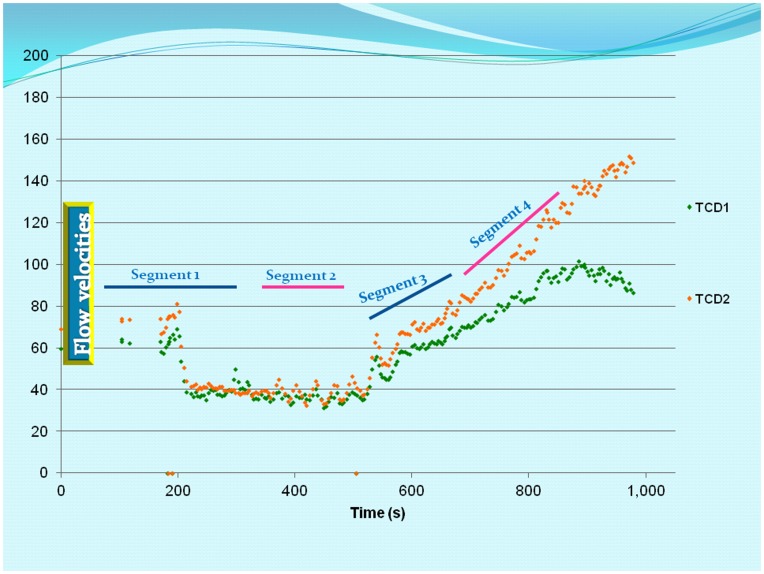
Bilateral middle cerebral artery blood flow velocities (Y-axis) against time in seconds (X-axis) in one subject. All subjects followed similar trends. The records were divided into 4 segments; we analyzed segment 2 considered as baseline (taken before the CO_2_ induced hyperventilation phase) and segment 4 taken as the response of the cerebral circulation to increasing blood CO_2_ levels during Duffin’s hypoxic ventilatory response test (orange, TCD 1 = left MCA flow velocities; green, TCD 2 = right MCA flow velocities.

However, significant changes in cerebral blood flow responses to rising CO2 levels induced by “Duffin’s rebreathing test” were recorded (Fig. 2).

### Cerebrovascular Responses to Rising Carbon Dioxide

**Figure 3:**
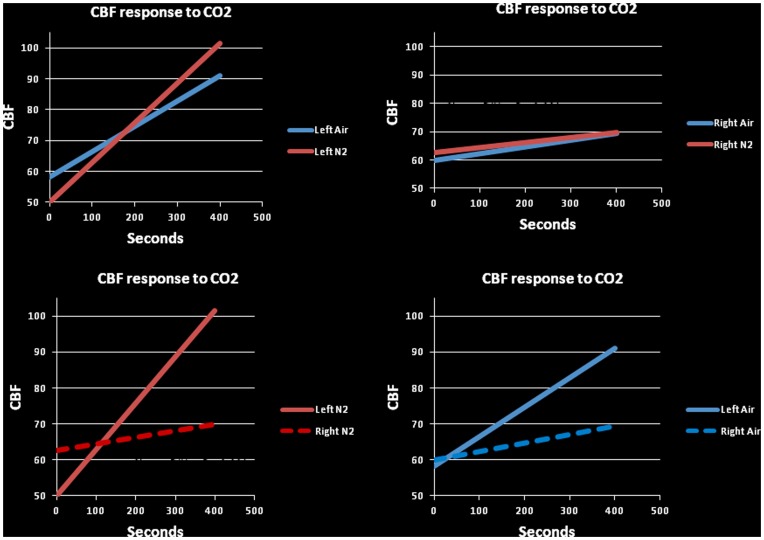
Predicted responses to carbon dioxide of the middle cerebral blood flow velocities under the two experimental conditions. The slopes of the fitted lines in all subjects were significantly different and the response was significantly enhanced in the left middle cerebral artery as compared to the right. (P<0.001, left and right upper panels). The responses of the cerebral circulation to increasing CO_2_ levels during skin hypoxia and skin normoxia were significantly enhanced on the left compared to the right (P<0.001, left and right lower panels). Additionally, both were significant compared to the baseline (P<0.001).

To explore the effects of skin hypoxia on the transcranial Doppler records we examined predicted middle cerebral artery (MCA) responses to rising carbon dioxide levels under both experimental conditions; skin normoxia and skin hypoxia Fig. 3. For this analysis we checked that the time series especially in segment 4 (the rebreathing phase) was stationary. We found significant differences in the fitted lines (P<0.001) between skin normoxia and skin hypoxia conditions. There also was a significant reduction in the response of the right MCA compared to the left flow velocities (P<0.001) (see also (Fig. 2).

### Autonomic Nervous System (ANS) Function

A number of ANS function tests could be performed using the available beat to beat recordings of blood pressure and heart rate obtained from the MCA flow velocities. We used power spectra of blood pressure from these recordings to show that there were no significant differences using the left MCA flow velocities under skin normoxia or hypoxia *(Fig. S1)* **.**

**Figure 4:**
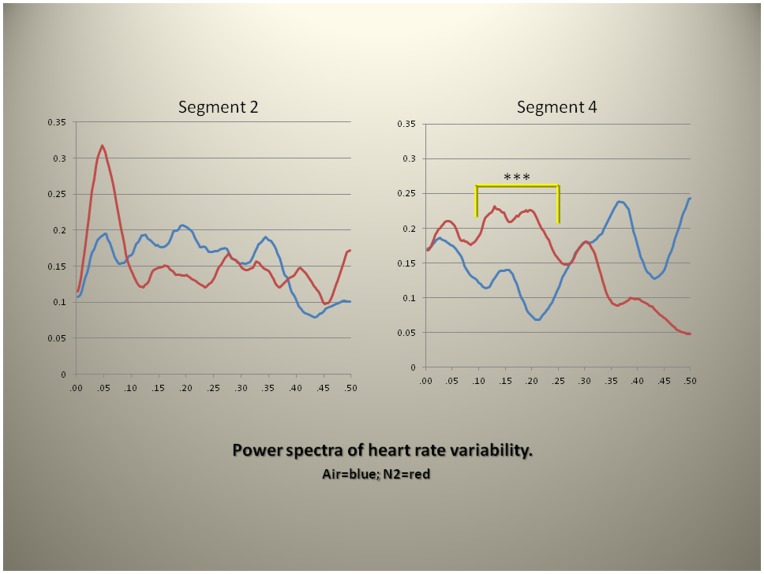
Power spectra of heart rate variability under two experimental conditions (skin normoxia = blue line; skin hypoxia = red line) during baseline (segment 2) and rebreathing (segment 4). A statistically significant difference in power of the low power segment (between 0.06–0.27 Hz, P<0.001^***^) was present. The frequency band selection was based on statistical considerations and not on International standards that define the low frequency bands for purposes of ANS control of cardiovascular function. Y-axis = Spectral power; X-axis = Frequency/2π.

However, power spectra obtained from heart rate variability of the baseline (segment 2) and during rebreathing (segment 4) of Duffin’s test of the hypoxic ventilatory drive showed a significant increase in low frequency power (between 0.06–0.27 Hz, P<0.001). (Fig. 4).

**Figure 5:**
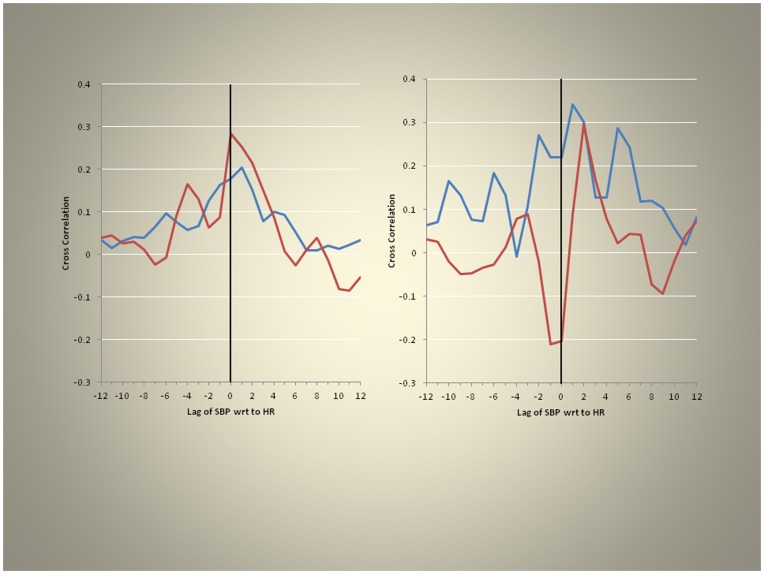
Cross correlation function (normoxia = blue; hypoxia = red). Under baseline conditions, with air in the bag, SBP followed heart rate with lag = 1, left panel, whereas during rebreathing (segment 4) SBP followed heart rate with a longer lag of 2, right panel. With the skin hypoxic these relationships of blood pressure and heart rate were further disrupted.

To assess brain blood flow homeostasis which depends largely on heart rate and blood pressure we performed cross correlation analyses of these parameters under skin hypoxia and skin normoxia (Fig. 5). Heart rate followed systolic blood pressure with a lag of one under base line conditions (segment 2) but during rebreathing (segment 4) heart rate followed systolic blood pressure with a lag of two while the skin remained normoxic. Under skin hypoxia, however, these relations were altered; for segment 2 the two parameters were synchronous and for segment 4 the lag was three. Thus we found that the normal relationships between heart rate and blood pressure changes with skin hypoxia were altered. This supports the notion that ANS function is affected by regional skin hypoxia.

**Figure 6:**
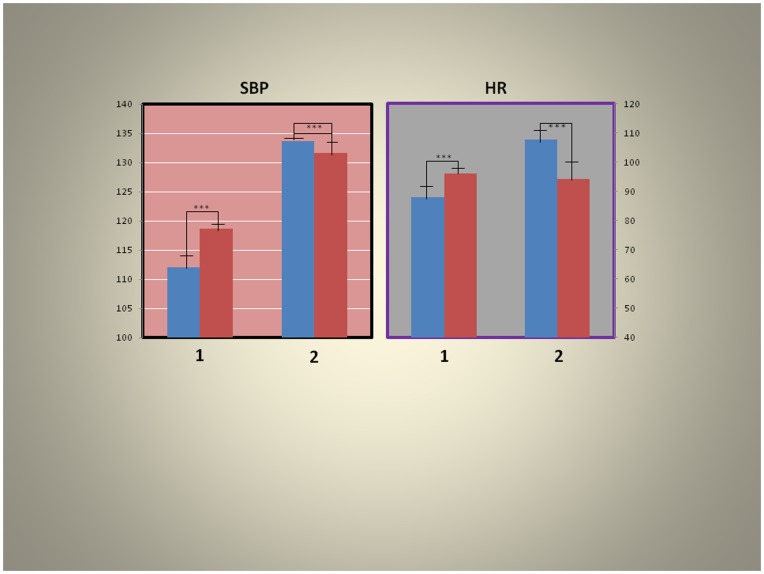
Effects of skin hypoxia on systolic blood pressure (SBP) and heart rate (HR) (blue = normoxia, red  =  skin hypoxia; left = baseline, right  =  during rebreathing). Note that there were statistically significant increases (P<0.0001) in systolic blood pressure with skin hypoxia (1) at baseline but this was converted to a statistically significant decrease (P<0.0001) during rebreathing (2), left panel. The changes were similar for heart rate during the two condition of the experiment, right panel (analysis of records from surface recording devices such as ear oximeter).

We appraised the effects of skin hypoxia on systolic blood pressure and heart rate as gleaned from the beat to beat records of the MCA flow velocities (Fig. 6). There were significant increases (P<0001) in systolic blood pressure with the skin under normoxic conditions during the baseline (segment) but this was changed to a significant (P<0001) decrease during rebreathing (segment 4). The changes were similar for heart rate during the two conditions of the experiment.

These results are consistent with an impairment of ANS control during hypoxia such that the relationship of blood pressure and heart rate were disturbed mimicking the results of the cross correlation analyses (Fig. 5).

**Figure 7:**
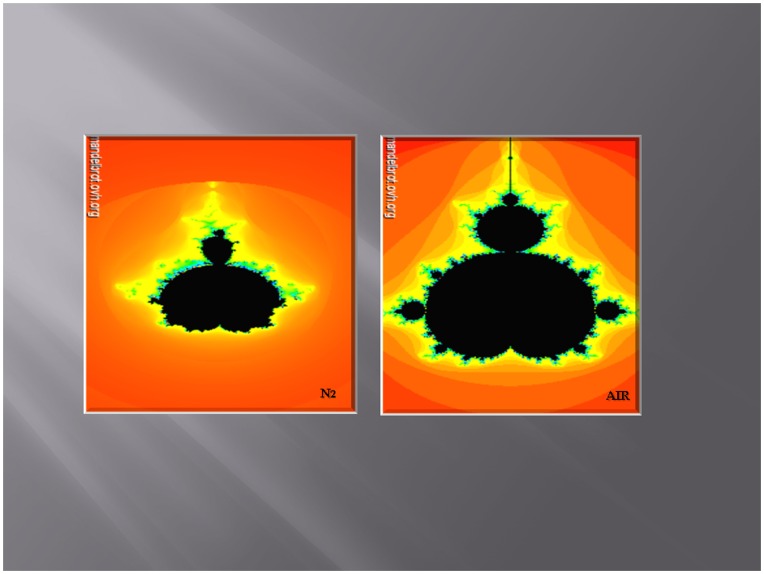
Mandelbrot set under skin normoxia (air) and hypoxia (N_2_) derived from the low power spectrum on the X-axis and the high power spectrum on the Y-axis of heart rate variability in one subject. (Mandelbrot.ovh.org). Note the width of the fractal illustration from the same individual under skin hypoxic condition is much reduced.

To illustrate the complex relationships of the low and high power spectra of the heart rate variability and skin hypoxia we used fractal analysis. We show these relationships using Mandelbrot fractal sets (Fig. 7). The set, derived under skin hypoxia, is changed in contour such as to reduce the width and height of the points derived from the data obtained from the cerebral circulation also consistent with ANS dysfunction.

## Discussion

In normal humans, skin hypoxia, in the absence of systemic hypoxia, modulates autonomic control of cardiovascular function and flow velocities in the cerebral circulation as gleaned from Doppler records. This occurs without recordable changes in surface monitors of blood pressure, skin temperature, heart rate or oxygen saturation.

Our findings implicate the human skin in the acute responsiveness to hypoxia. But the skin may be crucial for long term adaptation to life in ambient hypoxia of altitude; in this setting it may also play a role in maladaptive syndromes found in highlanders especially in the high Andes [5].

The skin covers the entire body; it offers a barrier to injury and maintains body integrity. In some vertebrates, such as amphibians and mice, it plays important roles in adaptation to the environment and to ambient hypoxia. [4].

In modern humans the skin is usually covered by clothing which augments skin barrier function and supplements its responsiveness to environmental change. Humans can use current fashions to create an individual’s microclimate; this adds to the repertoire of human adaptability to acute environmental changes and extends the limits of what they can do in extreme environments.

Our observations have relevance to fashions in clothing and to contemporaneous life styles. The effects of skin coverings which may induce regional skin hypoxia with subsequent modulation of the cerebral circulation and ANS function may also become relevant in conditions associated with systemic hypoxia such as lung disease, cardiac failure, in altitude sojourn and long term survival at great heights.

Near complete skin coverings used in some sports (swimming and diving) and in military operation of supersonic jets may impact the cerebral circulation and ANS function such that performance could be impaired, notwithstanding the supply of adequate systemic oxygenation through masks or other means.

Given the effect of skin hypoxia in animals we first thought to assess the hypoxic ventilatory drive in our human subjects. We studied this using Duffin’s rebreathing test [6]. We found no differences in the two conditions of the experiment. Because this test allows for the functional assessment of central and peripheral chemoreceptors we concluded that the changes we observed in the cerebral circulation and in the autonomic nervous system’s control of cardiovascular function could not be related to alterations in chemoreceptor activity.

The methods used here to investigate the effects of skin hypoxia on cerebrovascular and cardiovascular functions allowed beat to beat analysis of middle cerebral artery flow-velocities in response to acute changes in end tidal carbon dioxide (Fig. 2), and facilitated detailed analysis of ANS function during the two conditions of the experiment.

We directed our attention to the cerebral circulation, specifically, to the middle cerebral artery (MCA) flow velocities as recorded by the placement of our Doppler probes on the head of the subjects. We used statistical methods to predict flow velocities in the MCA and found that the responses of the flow velocities, during rising CO_2_ levels induced by the rebreathing, part of the tests of hypoxic ventilatory drive, were larger on the left, blood flowing to the dominant hemisphere, compared to the right (all subjects were right handed implying left hemisphere dominance) (Fig. 3).

Rising end-tidal CO_2_ (*P*_ET.CO2_) levels induced, not surprisingly, significant increases in the slopes of the predicted flow velocities but these were also significantly larger on the left; the dominant hemisphere (Fig. 3). This suggests that neural activity might be implicated in the differential vascular responses between the right and left sides.

Different responses on the two sides of the body in ANS control of the peripheral circulation have previously been reported [7].Our finding of this asymmetry in the cerebral circulation implies that the dominant hemisphere might require more blood for optimal function, or possibly this may reflect asymmetry in tissue mass, the left side requiring more blood flow to supply a larger volume of tissue.

We next examined blood pressure during the two conditions of the experiment. The power spectra of the systolic blood pressure obtained from the MCA beat to beat records showed no differences between baseline and segment 4 (records from the rebreathing phase of the experiment) and no differences between the two conditions of the experiments (skin normoxia and skin hypoxia) implying that blood pressure is not the prime mechanism of change, in this setting, but that other factors maintain homeostasis. By contrast similar analysis of heart rate variability (also a time series analysis) from the same records showed a significant increase in low power of the spectrum during skin hypoxia (Fig. 4).

A paradigm of autonomic neurovascular control has been that the low power of heart rate variability time series reflects the drive of the sympathetic nervous system to the cardiac pacemaker. Now this paradigm has been thrown into doubt [8]. The more recent view is that the low power of the spectrum reflects baroreflex function independent of the sympathetic drive to the cardiac pacemaker [8].

Activation of the sympathetic nervous system during acute ambient hypoxia at altitude in humans has been well recognized [9]. However, in the absence of systemic hypoxia, as in these experiments, sympathetic activation by regional skin hypoxia alone implies different mechanisms for this activation, independent of systemic hypoxic input and consistent with the absence of differences in the hypoxic ventilatory drive during the two conditions of the experiment. Moreover, the recent attribution of low frequency power to baroreflex function rather than sympathetic activation (see reference [8]) is consistent with our discovery that human regional skin hypoxia alters baroreflexes as assessed by cross correlations (see below).

Baroreceptor reflexes control circulatory responses to changes in posture and maintain adequate blood flow to tissues, in conditions such as recumbence or upright posture. This insures proper blood flow to the brain and prevents syncope (a sudden loss of consciousness). Baroreflex function is controlled by the ANS and it is impaired in various ANS disorders such as diabetic neuropathy; it can be assessed using spectral and cross spectral analysis of heart period and systolic blood pressure time series, using transfer function [10]. Normally, systolic blood pressure follows heart rate. In this study this time-relationship was retained. However, during rebreathing this relationship was altered by skin hypoxia (Fig. 5). These results are consistent with significant modulation of baroreceptor function by skin hypoxia. This might negatively affect cerebral blood flow in conditions of stress to the cerebral circulation such as hyperthermia and predispose to syncope when the skin is covered by clothing because during increased blood flow demands there is normally cardiac acceleration. Our results imply that skin hypoxia delays this response and thus impairs the proper timing of homeostatic blood pressure regulation.

Systolic blood pressure (not the power spectra of systolic blood pressure, see above) as recorded from MCA flow velocities increased during skin hypoxia (Fig. 6) but this significantly decreased during rebreathing suggesting that the vasodilatation observed in the cerebral circulation may have been more widespread, affecting also other organs. This matches the increased lag observed in systolic blood pressure (systolic blood pressure follows heart rate with a lag = 2, (Fig. 5) on cross-correlation) supporting evidence for altered baroreceptor function during skin hypoxia.

Heart rates (Fig. 6) show similar changes to systolic blood pressure. The decrease in heart rate with skin hypoxia may also reflect baroreceptor impairment; normally the heart accelerates in the presence of vasodilatation to maintain appropriate flow to organs especially the brain.

Fractals, a term coined by Benoit Mandelbrot, are often used to illustrate complex relationships of multiple measures such as the contour of mountains and the lengths of coast-lines. In biology they are used to show the relations of the ¾ scaling (M^¾^) of body size, of cellular metabolism, of the circulation, of life-span and heart rate [11]. Our illustrations of heart rate variability using Mandelbrot sets (Fig. 7) support the increase in low power found on spectral analysis of heart rate variability during hypoxia of the skin (Fig. 4). They show a loss of symmetry and decrease in scale also consistent with the increase in systolic blood pressure during skin hypoxia.

How could these widespread effects on the cerebral circulation and ANS function, induced by regional skin hypoxia be explained? We suggest that the release of nitric oxide (NO) from hypoxic skin keratinocytes [1] is the most likely and most important agent of change.

The skin has a role in modulating vascular responses to environmental changes in numerous vertebrates. In mammals it is also essential in adaptation to low oxygen levels [4]. In mice, NO release, induced by skin hypoxia due to activation of HIF (hypoxia inducible factors) controls the cutaneous circulation [2] our results imply that in humans the release of NO during skin hypoxia affects the cerebral circulation and especially ANS function. To our knowledge, such effects have not previously been noted in animal studies.

Erythropoietin (EPO) is known to preserve proper oxygenation of the blood. Acute responses to hypoxia are thought to involve EPO and the skin has been implicated in this response in mice [2]. An attempt to replicate these findings in humans has been unsuccessful [12]. In the human skin regional hypoxia induces its distant effects primarily by affecting the cerebral circulation and maintaining homeostasis through modulating ANS reactivity. In this setting, because of the upright posture, the cerebral circulation is threatened and rapid rather than, the somewhat, delayed effect of EPO is required to maintain homeostasis.

Altitude is not conducive to long term human survival; nevertheless, permanent settlements are reported at 5100 meters above sea level [13]. The responses of the cerebral circulation to NO induced vasodilatation have been found to be a measure of adaptability for survival at altitude [14]. Not surprisingly adequate blood supply to the brain in ambient hypoxia is important for adaptation to hostile environments and this is promoted by NO-induced vasodilatation. Thus our results imply that skin hypoxia may contribute to human adaptability to life at altitude.

Reprogramming of adult human fibroblasts obtained from skin biopsies is now common place and keratinocytes can be harvested that retain the specific genetic profiles of the donors’ material [15]. Adaptation to altitude in highlanders is heterogeneous for example in East Africans it is better than in Tibetans and Andeans are thought to be the worst adapted to the inescapable hypoxia in which they live [16]. Thus keratinocytes obtained from induced pluripotent stem cells, reprogrammed from these highland populations, could determine if the human skin contributes to an altitude adapted life style.

The physiological study of homeostatic regulation of responses to hypoxia in humans was, until now, based almost entirely on the control of respiration and blood oxygen content. Here we suggest a “paradigm shift” [17] in the control of the oxygen supply to tissues which enlists blood flow, as well as blood oxygen content in maintaining homeostasis and implicates regional hypoxia of the skin as an additional important player in human oxygen homeostasis.

Further studies are needed, however, to assess the magnitude of this paradigm shift in subjects with varying lifestyles, occupational hazards and habitats.

### Study Limitations

This study was carried out in normal, young, subjects, however, it did not allow us to fully assess the implications for various age groups and life styles nor can we recommend any changes that might mitigate the effects of skin hypoxia on cerebral blood flow or ANS function.

## Methods

The study was approved by the Ethics Review Board of the University Health Network, Toronto, Canada. Informed written consent was obtained from all participants. This study has been conducted according to the principles expressed in the Declaration of Helsinki. There were nine healthy males and three healthy females. All were between 20 and 40 years of age (mean±SD 26.8±2.97).

### Protocol

Chemorefelex control of breathing tests.

*Pre-test instructions:*

- Light meal not less than 2 h before testing
- No consumption of cigarettes, alcohol or caffeine 12 hours to day of study
- No heavy exercise 12 hours prior to day of testing

#### Control condition

Between tests and during baseline conditions the subjects breathed room air. During the tests subject were sitting in the plastic body bag which bared skin access to room air and was sealed around the neck, they were breathing room air via a face mask on a breathing circuit The body bag was connected to gas cylinders containing 100% oxygen and 100% nitrogen, which alternately filled the body bag according to the protocol. Flow of gases into the bag was ∼ 10 L/min. For detailed description of testing protocol see Text S1.

We used a modification of Read’s rebreathing test [18], “The Duffin rebreathing method” [19] to compare ventilatory and cerebrovascular responses to hypoxia during two conditions: 1. When the plastic bag encasing the whole body was filled with air (skin normoxia) or 2. The bag was filled with pure nitrogen (skin hypoxia).

The test order was randomly assigned. The subjects breathed room air before, in-between and after the rebreathing tests. All subjects were seated comfortably upright during the rebreathing tests and wore a finger pulse oximeter probe (Text S1).

### Testing Protocol

#### Rebreathing test analysis

Breath-by-breath 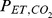
values were plotted against time and fitted with a least squares regression line. The plots were fitted and divided into two segments which were separated by a breakpoint, the ventilatory response threshold, which is defined here as the 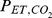 where ventilation increase in response to rising 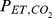. (Text S1).

### Statistical Analysis and Cerebrovascular Responses to Rising Carbon Dioxide

To determine the effects on the cardiovascular system and autonomic nervous systems while nitrogen was circulated in the body-bag, we compared the results to that when the bag was filled with air, we did the following:

1. Defined time segments (labeled 2-baseline and 4-the rising phase of cerebral blood flow in response to increasing CO2 levels during the rebreathing test): while air was in the bag and again when nitrogen was in the bag (Fig. 2).
2. The outcome variables were right and left brain CBF (bilateral MCA flow velocities), Systolic BP, and HR. The rate of change (slope) over time in CBF in each time segment was compared using Repeated Measures (RM) ANOVA with condition (nitrogen and Air), side, and time as repeated factors. Time was treated as a linear factor in order to obtain slope comparisons. Results were verified by graphical methods.
3. To determine autonomic control of cardiac rhythms, we de-trended and standardized the time series in segment 4 (during each intervention) and then did spectral analysis using the appropriate SAS (version 9.2) procedures. The low, medium and high frequency band spectral content of the resulting spectra were defined according to accepted standards in autonomic cardiovascular clinical usage. We computed comparisons using t-tests.
4. Systolic BP, and HR where compared in terms of means by RM ANOVA similar to the analysis in item 2, above.
5. Cross correlation of systolic BP and HR time series were used to determine lags under the two conditions prevailing in the bag (nitrogen and Air) to assess baroreceptor function (Text S1).

### Fractal Analysis

Data obtained from the power spectra of heart rate variability were inputted to the Mandelbrot set-online generator by Dawid Makiela© (Mandelbrot.ovh.org).The low power was on the X-axis and the high power of the power spectrum was on the Y-axis (Text S1).

## Supporting Information

Figure S1 (DOCX)

Text S1 (DOCX)
