# Supplementary material for: Human Skin Hypoxia Modulates Cerebrovascular and Autonomic Functions
Source: PLoS One. 2012 Oct 8;7(10):e47116. doi: 10.1371/journal.pone.0047116 (PMC3466185; doi:10.1371/journal.pone.0047116)
Supplement: Figure S1 — (DOCX) [file pone.0047116.s001.docx]

**SOM Fig 1. Power spectra obtained from blood pressure.**

Recordings from the left middle cerebral artery; there were no significant differences during the two conditions of the experiment. Left panel=segment 2; right panel=segment 4. Blue=skin normoxia; red=skin hypoxia.
