# Supplementary material for: Human Skin Hypoxia Modulates Cerebrovascular and Autonomic Functions
Source: PLoS One. 2012 Oct 8;7(10):e47116. doi: 10.1371/journal.pone.0047116 (PMC3466185; doi:10.1371/journal.pone.0047116)
Supplement: Text S1 — (DOCX) [file pone.0047116.s002.docx]

# Supplementary on line information Text S1

**Test protocol:**

- Initially subjects were sitting at rest with room air flowing into the body bag for at least 5 min. Control values were recorded.
- The order of N_2_ or O_2_ flowing into the body bag was chosen by flipping a coin. The subject but not the investigator was blinded to the result of the toss. The flow rates were the same for all gases.
- Next subjects performed the tests to determine the hypoxic ventilatory drive.
- After the completion of the test a rest period of 15 minutes was allowed while subjects were breathing room air. Then the test was repeated with a different gas mixture in the bag. After the second test the subject was again exposed to room air.
- Subjects breathed via the Duffin Rebreathing system face mask [1]. During testing subjects were seated on a comfortable chair in a quiet room and fitted with the face mask. The mask was connected to a three-way, manually operated valve (Hans Rudolph Inc., Kansas City, MO, USA) via a mass flow sensor (AWM720P1 Airflow, Honeywell; Freeport, Illinois, USA). One way of the three-way valve was left open to room air and the other to a 2-metre length of rebreathing tubing. This rebreathing tubing was supplied with gas from a programmable gas mixing system (Respiract™, TRI; Toronto, Canada) at the 3- way valve and left open to room air at its distal end. This setup allowed us to quickly and easily switch the subject between breathing room air and mixed gas.
- The rebreathing bag had a volume of 5 l and an inlet for oxygen. It was primed with a CO_2_–O_2_ mixture to ensure appropriate equilibration at the beginning of a rebreathing test. Inspired and expired partial pressures of CO_2_ and O_2_ were sampled at the mouth and monitored throughout the test using gas analyzers (Models 17630 and 17625, VacuMed). Ventilation was measured using a flow transducer (AWM720P1, Honeywell). Continuous data were imputed to a computer via an analog-to-digital converter (DAQCard-6024E, National Instruments, Austin, TX, USA). A specially written program (Lab VIEW, National Instruments, Austin, TX, USA) analyzed the data to provide a file of breath-by-breath
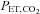
, end-tidal
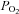
 (
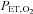
) and ventilation. In addition, the program operated a solenoid valve controlling the flow of oxygen to the rebreathing bag to maintain
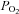
 at 150 mmHg (hyperoxic) or at 50 mmHg (hypoxic) during rebreathing. The
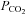
 and
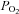
 analyzers were calibrated using gas from cylinders of analyzed medical grade compressed gases, and ventilation was calibrated using a 3 l calibration syringe (Model R5530B, Vacumed, Ventura, CA, USA) (series 5530, Hans Rudolph Inc., Kansas City, MO, USA) . Portable pulse oximeters (Nonin Onyx II 9550, Nonin Medical Inc, Plymouth MN, USA) were used to measure arterial oxygen saturation and heart rate.
- Each rebreathing test began with a 5 min hyperventilation of ambient air, with subjects coached to maintain their
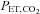
 between 19 and 25 mmHg. Subjects then exhaled completely and were switched to the rebreathing bag where they took three deep breaths to facilitate rapid equilibration of
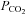
 in the bag, lungs and arterial blood to that of mixed venous blood. This equilibration was verified by observing a plateau in the end-tidal
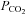
, and was a prerequisite for continuing the test. The rebreathing test ended when ventilation exceeded 100 l min^−1^, or
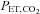
 exceeded 70 mmHg, or if discomfort occurred. The testing environment was quiet, with minimal distractions. Subjects were instructed to relax and close their eyes during the rebreathing experiments.
- Subjects were fitted with bilateral Doppler probes to insonate the middle cerebral arteries. These were kept in place throughout the rebreathing tests by a head band to maintain their position constantly throughout each test.

## Mandelbrot set

The Mandelbrot set consists of a number of points; it does not represent anything more than these points. The images generated are not just in the Mandelbrot set but are also outside the points of interest and are colored in such a way as to represent how long it took to decide that a point was not in the set.

A Mandelbrot set is generated by a mathematical operation such that for each point c in some region of a complex plane the X value represents the "real" part of a complex number and the Y value represents the "imaginary" part of the complex number. The operation consists by repeating the following equation: z_n+1_ = z_n_^2^ + c (where z_0_ = 0). If the absolute value of z_n_ remains within the bounds, then the point c is in the Mandelbrot set. If, z_n_ goes to infinity, then the point remains outside the bounds of the set.

The colors of the set are dependent on the number of calculations, here 100 iterations. The Mandelbrot set is not a simple shape, but is irregular. The set is named after Benoit Mandelbrot, who coined the term "fractal" for these irregular shapes.

### Statistical analyses

To determine to effects of N_2_ in the body-bag on the cardiovascular and autonomic nervous systems, to that when the bag was filled with air we did the following:

1. Defined two time segments (labeled 2, the baseline and 4, the rising phase of the blood flow velocities due to the increasing CO_2_ levels during the rebreathing phase of the hypoxic ventilatory drive test): while air was in the bag and again when N_2_ was the prevailing condition in the bag.

2. The outcome variables were right and left brain CBF (bilateral MCA flow velocities), Systolic blood pressure, and heart rate.

The rate of change (slope) over time in CBF in each time segment was compared using Repeated Measures (RM) ANOVA with condition (N2 and Air), side, and time as repeated factors. Time was treated as a linear factor in order to obtain slope comparisons. Results were verified by graphical methods.

3. To determine autonomic control of cardiac function, we de-trended and standardized the time series in segment 4 (during each intervention) and then did spectral analysis using the appropriate SAS (version 9.2) procedures. The low, medium and high frequency band spectral content of the spectra were defined according to accepted standards in autonomic cardiovascular clinical usage and computed, using formulas from Priestley [2] We computed comparisons using t-tests.

4. Systolic BP, and HR where compared in terms of means by RM ANOVA similar to the analysis in item two, above.

5. Cross correlation of systolic blood pressure and heart rate time series were used to determine lags under the two conditions (N_2_ and Air) to assess baroreceptor function.

### Cross correlation analysis

### We performed beat-to-beat analysis of the signals from the flow velocities of the middle cerebral arteries by extracting the time series of successive values of R–R interval (RR) and systolic arterial pressure (SBP) while the bag was filled with air or nitrogen. An autoregressive monovariate model was fitted to each RR and SBP time series, and the powers associated to each oscillatory component were quantified by computation of the residuals. Two main oscillatory components were detected: one at low frequency (LF; ∼0.1Hz) and one at high frequency (HF is related to the respiratory rate). We also performed cross-spectral analysis of RR and SBP, using a bivariate autoregressive model. This technique quantifies the frequency-related squared coherence, phase shift, central frequency and transfer function gain between two variables at a given frequency. Since this method provides a smooth estimate of the true cross-spectra, discrete values of phase shift, central frequency and transfer function gain between RR and SAP were taken at the frequency corresponding to the highest coherence value, where the estimate error is minimal.

We accepted the cross-spectral data for analysis only when coherence values were above 0.5 (possible range, 0–1) because this is considered to indicate a statistically significant linear correlation between the two signals. A negative phase shift indicates that changes in SBP precede changes in RR [3].

### References

1. Battisti-Charbonney A, Fisher JA, Duffin J (2011). Respiratory, cerebrovascular and cardiovascular responses to isocapnic hypoxia. [Respir Physiol Neurobiol.](http://www.ncbi.nlm.nih.gov/pubmed/21939786) 179:259-26
2. Priestley M (1981) Spectral Analysis and Time series page 427 and ff. Vol. 1 Academic Press San Diego, Ca.
3. Gulli G, Claydon VE, Slessarev M, Zenebe G, Gebremedhin A. (2007) Autonomic regulation during orthostatic stress in highlanders: comparison with sea-level residents. [Exp Physiol.](http://www.ncbi.nlm.nih.gov/pubmed/17138623) 92:427-35.
